# Understanding the Effects of Trenbolone Acetate, Polyamine Precursors, and Polyamines on Proliferation, Protein Synthesis Rates, and the Abundance of Genes Involved in Myoblast Growth, Polyamine Biosynthesis, and Protein Synthesis in Murine Myoblasts

**DOI:** 10.3390/biology12030446

**Published:** 2023-03-14

**Authors:** Laura A. Motsinger, Lillian L. Okamoto, Nikole E. Ineck, Brynne A. Udy, Christopher L. Erickson, Youssef Harraq, Caleb C. Reichhardt, Gordon K. Murdoch, Kara Jean Thornton

**Affiliations:** 1 Department of Animal, Dairy and Veterinary Sciences, Utah State University, Logan, UT 84322, USA; 2 Department of Animal Sciences, Washington State University, Pullman, WA 99163, USA

**Keywords:** anabolic hormones, estradiol, myoblast, polyamine, proliferation, protein synthesis, skeletal muscle growth, trenbolone acetate

## Abstract

**Simple Summary:**

Anabolic hormones, such as estradiol and testosterone, are known to promote skeletal muscle growth in many different mammalian species. However, there are several different concerns with using anabolic hormones to improve or remedy skeletal muscle growth. As such, natural growth-promoting alternatives to anabolic hormones are needed. Previous research suggests that one mechanism through which anabolic hormones improve proliferation and protein synthesis within skeletal muscle is through modulation of the polyamine biosynthetic pathway. Polyamines are naturally occurring amino acid derivatives that are known to be potent stimulators of growth. As such, the purpose of this study was to examine the effects of anabolic hormones, polyamine precursors, and polyamines, relative to proliferation, protein synthesis rates, and messenger RNA expression in cultured murine myoblasts. The results demonstrate that anabolic hormones, polyamine precursors, and polyamines increase proliferation and anabolic hormones increase protein synthesis rates. Furthermore, polyamines and their precursors alter expression of the genes involved in polyamine biosynthesis, proliferation, and protein synthesis. However, additional research is needed to further investigate the relationship between anabolic hormones and polyamines relative to skeletal muscle growth to determine if polyamines and their precursors can be utilized as natural growth-promoting alternatives to anabolic hormones.

**Abstract:**

Research suggests that androgens increase skeletal muscle growth by modulating polyamine biosynthesis. As such, the objective of this study was to investigate effects of anabolic hormones, polyamine precursors, and polyamines relative to proliferation, protein synthesis, and the abundance of mRNA involved in polyamine biosynthesis, proliferation, and protein synthesis in C2C12 and Sol8 cells. Cultures were treated with anabolic hormones (trenbolone acetate and/or estradiol), polyamine precursors (methionine or ornithine), or polyamines (putrescine, spermidine, or spermine). Messenger RNA was isolated 0.5 or 1, 12, or 24 h post-treatment. The cell type had no effect (*p* > 0.10) on proliferation, protein synthesis, or mRNA abundance at any time point. Each treatment increased (*p* < 0.01) proliferation, and anabolic hormones increased (*p* = 0.04) protein synthesis. Polyamines increased (*p* < 0.05) the abundance of mRNA involved in polyamine biosynthesis, proliferation, and protein synthesis. Treatment with polyamine precursors decreased (*p* < 0.05) the abundance of mRNA involved in proliferation and protein synthesis. Overall, C2C12 and Sol8 myoblasts do not differ (*p* > 0.10) in proliferation, protein synthesis, or mRNA abundance at the time points assessed. Furthermore, anabolic hormones, polyamines, and polyamine precursors increase proliferation and protein synthesis, and polyamines and their precursors alter the abundance of mRNA involved in growth.

## 1. Introduction

Muscle fiber number in mammals is predominantly fixed at birth and, therefore, post-natal muscle growth occurs almost exclusively through hypertrophy of existing muscle fibers [1]. In times of muscle growth, injury, or regeneration, quiescent muscle satellite cells become activated, which is marked by a decreased abundance of paired box transcription factor 7 (*Pax7*) [2] and Sprouty 1 (*Spry1*) [3,4], and differentiate into myoblasts [5,6]. Myoblasts then proliferate, marked by an increase in mitogen-activated protein kinase (*MapK*) abundance [7], and fuse with existing muscle fibers to support hypertrophy during post-natal growth [5,6]. Skeletal muscle tissue is highly plastic and is continually being remodeled through a balance of both protein synthesis and protein degradation [8]. Muscle protein turnover is necessary to maintain muscle mass [8]. Ultimately, for skeletal muscle growth to occur, muscle protein synthesis, especially synthesis of the contractile myofibrillar protein fraction, must be greater than muscle protein degradation [8], which is indicated by an increased abundance of the mammalian target of rapamycin (*mTOR*) [9] and eukaryotic translation initiation factor 2B subunit epsilon (*eIF-2Bε*) [10], among other genes. 

Androgens have become molecules of interest relative to skeletal muscle growth in both humans and livestock. In humans, testosterone can be used as a remedy for conditions such as sarcopenia, i.e., muscle atrophy associated with natural aging processes [11,12,13]. In livestock species, trenbolone acetate (a testosterone analog, TBA) is commonly administered to beef animals in the US in the form of anabolic implants to improve growth and production efficiency [14]. Studies have established that administration of androgens results in increased skeletal muscle growth in many species including humans [15,16], mice [17], and cattle [18], to name a few. However, the observed effects of androgens on proliferation, differentiation, and protein synthesis are inconsistent throughout the literature. Androgens have been shown to both stimulate [19] and have no effect [20] on the proliferation of C2C12 murine myoblast cells and increase the proliferation of primary bovine satellite cells [21,22]. Additionally, previous research has demonstrated that the treatment of C2C12 cells with androgens has no effect on protein synthesis [20], but increases differentiation [19], and the treatment of bovine satellite cells with androgens results in increased protein synthesis [18,23]. However, the exact mechanism through which androgens increase growth of skeletal muscle is unknown. Past research suggests that one mechanism by which androgens, such as trenbolone acetate, and estrogens, such as estradiol-17β (E2), may improve growth is through an interaction with the polyamine biosynthetic pathway [22,24,25,26,27,28,29,30,31].

In the polyamine biosynthetic pathway, methionine (Met), ornithine (Orn), and arginine (Arg) are utilized as substrates to produce putrescine (Put), spermidine (Spd), and spermine (Spe), which are the three polyamines found in mammalian cells [24,32,33]. Ornithine decarboxylase (*ODC*) and S-adenosylmethionine decarboxylase (*AMD1*) are enzymes involved in the production of Put from Orn and decarboxylated adenosylmethionine, respectively [24,32]. Spermidine synthase is an enzyme involved in the production of Spd from Put or decarboxylated adenosylmethionine, and spermine synthase is an enzyme involved in the production of Spe from Spd [32]. Polyamines are naturally occurring amino acid derivatives with bioactivities that are essential for growth, cell proliferation, cell differentiation, and protein synthesis [32,34,35,36,37]. In skeletal muscle, polyamine biosynthesis increases during hypertrophy and decreases during atrophy [38]. Androgens are thought to regulate polyamine biosynthesis by increasing the expression of ODC and AMD1 [39,40]. However, the role of TBA in the polyamine biosynthetic pathway has not been well-characterized in skeletal muscle cells.

An improved understanding of the interaction between anabolic hormones, the polyamine biosynthetic pathway, and growth is essential for the development of alternative remedies for muscle atrophy in humans and also for the development of alternative growth-promoting technologies in cattle. As such, the goal of the present research was to better understand the effects of TBA, E2, polyamines, and polyamine precursors on proliferation, protein synthesis, and the mRNA abundance of genes involved in the polyamine biosynthetic pathway and skeletal muscle growth in proliferating and fused murine myoblasts. We hypothesized that TBA, E2, polyamines, and polyamine precursors would each increase proliferation, protein synthesis, and the mRNA abundance of genes involved in growth and that TBA would increase the abundance of *ODC* and *AMD1*. 

## 2. Materials and Methods

### 2.1. Culture of Murine Myoblasts

Sol8 and C2C12 mouse myoblast cell lines were obtained from American Type Culture Collections^®^ and grown according to manufacturer specifications. Sol8 cells are clonal cell lines obtained from the skeletal muscle of young mice. C2C12 cells are immortalized, non-cancerous clonal murine myoblast cells obtained from skeletal muscle sarcomas of adult mice. Cells remained stored in liquid nitrogen until resurrection. Cells were cultured in a growth medium that consisted of phenol-red free Dulbecco’s Modified Eagle Medium (DMEM) [41] with 10% fetal bovine serum (FBS) in 75 mL flasks and incubated at 37 °C with 5% CO_2_ in a water saturated environment [42]. Every 48 h, cells were washed twice with DMEM and fresh growth medium was added.

### 2.2. Treatment of Myoblast Cultures for Proliferation Assays

The cells were plated into 96-well plates for analysis of the proliferation rate, or into 12-well plates for mRNA isolation, at a density of 11,200 cells/cm^2^ with fresh growth medium and allowed 24 h to establish. Once cultures reached 70% confluency, they were washed twice with DMEM and treated. Cultures were treated with DMEM containing 1% FBS and 10 nM TBA, 10 mM Met, 8 mM Orn, 3 mM Put, 1.5 mM Spd, or 0.5 mM Spe. Control cultures for proliferation assays were treated with DMEM containing 1% FBS. The concentration of 1% FBS was chosen to create an environment that does not support growth in order to observe whether the treatments impact growth. Treatment concentrations for analysis of proliferation in the present study were selected based off of previous work from our laboratory group that demonstrated increased proliferation in bovine satellite cells [22]. Three separate replicates were completed for each cell type.

### 2.3. Treatment of Myoblast Cultures for Protein Synthesis Assays

The cultures were grown to approximately 70% confluency, plated, and given 24 h to establish. The cultures for analysis of protein synthesis were plated into 96-well plates, and the cultures for mRNA isolation were plated into 12-well plates, at a density of 11,200 cells/cm^2^. The cultures were subsequently grown to approximately 80% confluency, washed twice with DMEM, and induced to differentiate in DMEM containing 3% horse serum and 1.5% bovine serum albumin-linoleic acid (BSA-LA). Cytosine arabinoside was added to cultures 24 h after the addition of the differentiation media and remained on cultures for a minimum of 18 h to ensure that all proliferating cells were eliminated, resulting in a pure culture of myotubes. The cultures were then treated with serum-free media (SFM) containing DMEM, 10^−8^ M insulin from bovine pancreas, 250 µg fetuin from fetal bovine serum/mL, 100 µg BSA-LA/mL and/or one of the following: 10 nM TBA, 10 nM E2, 10 nM TBA + 10 nM E2, 10 mM Met, 8 mM Orn, 3 mM Put, 1.5 mM Spd, or 0.5 mM Spe. Control cultures received just SFM. The treatment concentrations used in the analysis of protein synthesis were selected based upon results from past studies completed by our laboratory that impacted the growth of bovine satellite cells [22,31]. Additionally, in the protein synthesis study, which was completed after the proliferation study, the E2 and TBA + E2 treatments were added because further review of past literature demonstrated evidence for a potential interaction between E2 and polyamine biosynthesis [29,30].

### 2.4. Analysis of Proliferation Rates

Proliferation assays were performed 21 h after treatment using a commercially available kit (DELFIA, PerkinElmer, Waltham, MA, USA) and following the manufacturer specifications and previously published methods [22]. In short, bromodeoxyuridine (BrdU) was diluted with DMEM to 1:100 and added to the cultures. The cultures were incubated with BrdU for 3 h to label proliferating cells. After incubation, the cells were fixed and anti-BrdU was added to the cultures followed by a 1 h incubation period. Results of the proliferation assay were analyzed via fluorescent detection on a BioTek Synergy H1 plate reader using the all-in-one microplate reader software, Gen 5 2.09 (BioTek Instruments, Winooski, VT, USA). The proliferation assays were run congruently with the mRNA isolation to establish the relationship between the mRNA abundance and proliferation rate.

### 2.5. Analysis of Protein Synthesis Rates

Protein synthesis assays were performed 3 h after treatment using a commercially available kit (Click-iT Plus OPP Alexa Fluor™ 488 Protein Synthesis Assay Kit, Invitrogen, Waltham, MA, USA) following the manufacturer specifications. In short, O-propargyl-puromycin (OPP) was diluted with DMEM to 1:100 and added to the cultures. The cultures were then incubated for 3 h to allow for newly synthesized proteins to be labeled with OPP. After incubation, the cultures were fixed to the plate with 3.7% formaldehyde and permeabilized with 0.5% ethanol. The cultures were then dyed with NuclearMask™ Blue Stain and incubated for 30 min. Results of the protein synthesis assays were analyzed via fluorescence detection on a BioTek Synergy H1 plate reader using all-in-one microplate reader software, Gen 5 2.09 (BioTek Instruments, Winooski, VT, USA). The protein synthesis assays were run congruently with the collection of mRNA to relate transcript abundance with protein synthesis.

### 2.6. mRNA Isolation, Quantification, and cDNA Synthesis

An Absolutely RNA Microprep Kit (Agilent Technologies, Cedar Creek, TX, USA) was utilized to isolate total RNA from the cultures following the manufacturer’s protocol. Briefly, cell lysate was collected at three different time points for analysis relative to proliferation (0.5, 12 and 24 h post-treatment) or protein synthesis (1, 12, and 24 h post-treatment). These time points were based off of previous work completed by our research group that demonstrated changes in gene expression in bovine satellite cells [22,31]. In addition, these specific time points were chosen because changes in gene expression are likely to be observed prior to a gross change in proliferation or protein synthesis. The first lysate time point for analysis of proliferation and protein synthesis differed because the proliferation study was completed before the protein synthesis study and, in the proliferation study, there were few changes in mRNA at 0.5 h post-treatment. Therefore, in the protein synthesis study, the first lysate time point was performed later at 1 h post-treatment. Prior to each lysate collection, the cells were washed once with phosphate-buffered saline (PBS) solution and cell lysis buffer was added to the cultures, and cells were lysed with mechanical scraping. The cell lysate samples were stored at −80 °C until the mRNA isolation was performed. The cell lysate samples were vortexed and received an equal volume of 70% ethanol. Each sample was then centrifuged, filtered, subjected to a series of wash buffers, and the mRNA was then eluted. The quantification of mRNA was performed using a Take3 plate on a BioTek Synergy H1 plate reader using the Gen 5 2.09 all-in-one microplate reader software (BioTek Instruments, Winooski, VT, USA). The quality of mRNA was determined by analyzing the 260/280 ratio and all samples that had a ratio greater than 2.0 were considered acceptable. Acceptable RNA samples were treated with deoxyribonuclease (Ambion, Foster City, CA, USA) and then converted into cDNA using the high-capacity cDNA reverse transcription kit (Applied Biosystem, Foster City, CA, USA) as per the manufacturer’s protocol.

### 2.7. Quantitative Real-Time PCR

Real-time PCR quantification and the TaqMan MGB primer/probe system were used following previously described protocols [43,44] to evaluate the mRNA abundance. The primers and probes for the genes that were investigated are shown in Table 1 and were designed using Primer Express 3.0 software (Applied Biosystems, Waltham, MA, USA) [22]. The relative mRNA abundance of ribosomal 18S (*18S*, housekeeping gene [43,44]), *ODC*, *AMD1*, *Pax7*, *Spry1*, *MapK*, *mTOR*, and *eIF-2Bε* were evaluated using an ABI 7500 real-time PCR system (Applied Biosystems, Waltham, MA, USA). The genes analyzed in the present study were selected because of their role in satellite cell and myoblast growth [3,4,7,9,10] and polyamine biosynthesis [39,40].

### 2.8. Statistical Analysis

The effect of cell type on the proliferation rate, protein synthesis rate, and mRNA abundance at each specific time point (0.5 or 1, 12 or 24 h) was assessed using the MIXED procedure of SAS^®^ (version 9.4; SAS Institute Inc., Cary, NC, USA) where cell type, cell type × treatment and treatment served as fixed effects and plate and experiment number were included as random effects in the model. No effects (*p* > 0.10) of cell type × treatment were found relative to the proliferation rate or mRNA abundance in proliferating cultures. The cell type had no effect (*p* > 0.10) on the protein synthesis rates or abundance of mRNA (*p* > 0.05) at any of the time points assessed. Consequently, the final model was altered to include the fixed effect of treatment, and cell type, plate, and experiment number were included as random effects. The effect of each treatment relative to the control on the proliferation rate, protein synthesis rate, and mRNA abundance at each specific time point (0.5 or 1, 12 and 24 h) was analyzed using a series of contrast statements within the mixed procedure of SAS, with each treatment serving as the fixed effect and plate, experiment number and cell type as random effects. All proliferation and protein synthesis rate data are displayed as the least squares mean ± SEM with values representing the fold change of treated cultures relative to the control cultures (with a set value of 1.0). The gene expression data are presented as the relative mRNA abundance of each sample (calculated as 2^-relative threshold cycle (ΔCt)^) relative to the control value. A *p* ≤ 0.05 was considered significant and a *p* > 0.05 and *p* ≤ 0.10 was considered a tendency for significance.

## 3. Results

### 3.1. Effects of Cell Type on Proliferation, Protein Synthesis, and Relative mRNA Abundance

**Figure 1:**
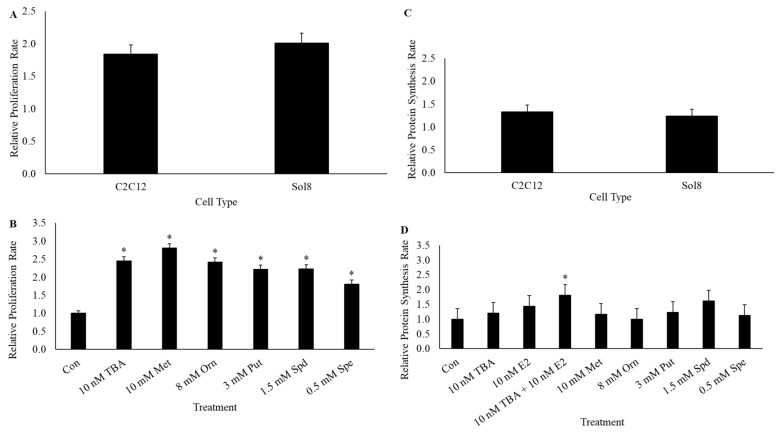
Effect of cell type (C2C12 or Sol8 myoblasts) or treatment on proliferation rates (**A**,**B**) and protein synthesis rates (**C**,**D**). Proliferation assay treatments included 1% fetal bovine serum (FBS, control, Con), 10 nM trenbolone acetate (TBA), 10 mM methionine (Met), 8 mM ornithine (Orn), 3 mM putrescine (Put), 1.5 mM spermidine (Spd), or 0.5 mM spermine (Spe). Protein synthesis assay treatments included serum-free media (SFM, control, Con) as a control and the other treatments were similar to those described in the proliferation assay, with the exception of the addition of 10 nM TBA + 10 nM E2. Values shown in A and B represent the relative proliferation rate of each treatment compared to control cultures and are presented as the least square mean ± SEM from three separate replicates of C2C12 cultures and three separate replicates of Sol8 cultures. Values from (**C**,**D**) represent the relative protein synthesis rate of each treatment compared to control cultures and are presented as the least square mean ± SEM from five separate replicates of C2C12 cultures and five separate replicates of Sol8 cultures. Treatments with a star (*) indicate differences (*p* ≤ 0.05) between treatment and control cultures in (**B**,**D**).

The cell type had no effect (*p* > 0.10) on the proliferation rate (Figure 1A). The cell type did not alter (*p* > 0.10) the mRNA abundance of genes involved in polyamine biosynthesis (*ODC* and *AMD1*) or genes involved in skeletal muscle growth (*Pax7*, *Spry1*, or *MapK*) at 0.5, 12, or 24 h post-treatment (Table 2).

The cell type had no effect (*p* > 0.10) on the protein synthesis rates (Figure 1C). Further, the cell type had no effect (*p* > 0.10) on the abundance of *ODC* or *mTOR* (Table 2) in fused cultures. The expression of *AMD1* tended (*p* = 0.06) to be increased in fused C2C12 cells 1 h after treatment when compared to fused Sol8 cells; however, no differences (*p* > 0.10) in *AMD1* abundance were observed between the different cell types at 12 or 24 h post-treatment (Table 2). The abundance of *eIF-2Bε* tended (*p* = 0.10) to be increased in fused Sol8 cells 24 h after treatment compared to fused C2C12 cells (Table 2). No differences (*p* > 0.10) in *eIF-2Bε* abundance were observed between the different cell types at 1 or 12 h post-treatment in fused cultures (Table 2).

### 3.2. Effects of Treatments on Proliferation and Protein Synthesis Rates of Murine Myoblasts

The treatment of C2C12 and Sol8 cells with 10 nM TBA, 10 mM Met, 8 mM Orn, 3 mM Put, 1.5 mM Spd, or 0.5 mM Spe each resulted in an increased (*p* < 0.01) proliferation rate when compared to control cultures (Figure 1B).

The treatment of cells with TBA + E2 increased (*p* = 0.04) the protein synthesis rate compared to the control cultures (Figure 1D). However, the treatment of cells with TBA, E2, Met, Orn, Put, Spd, or Spe had no effect (*p* > 0.10) on the protein synthesis rate when compared to the control cultures (Figure 1D). 

### 3.3. Effects of Treatments on the Relative mRNA Abundance of Genes Involved in Polyamine Biosynthesis

In the proliferating cultures, Spe increased (*p* = 0.02) the relative mRNA abundance of *ODC* 0.5 h post-treatment compared to the control cultures; however, no differences (*p* > 0.10) were observed 12 or 24 h post-treatment (Table 3). The relative mRNA abundance of *ODC* was increased (*p* = 0.05) 12 h post-treatment with Spd, but no differences (*p* > 0.10) in *ODC* expression in the proliferating cultures were observed 0.5 or 24 h post-treatment when compared to the control cultures (Table 3). No differences (*p* > 0.10) in the relative mRNA abundance of *ODC* in the proliferating cultures were observed 0.5, 12, or 24 h post-treatment when cells were treated with TBA, Met, Orn, or Put when compared to the proliferating control cultures (Table 3). Analysis of the effect of treatment on the relative mRNA abundance of *AMD1* showed no differences (*p* > 0.10) between the proliferating control cultures and the different treatments at 0.5, 12, or 24 h post-treatment (Table 3).

The treatment of fused cells with Orn tended (*p* = 0.06) to decrease *ODC* expression 1 h post-treatment when compared to the control cultures; however, no differences (*p* > 0.10) in *ODC* expression were observed between the control and treatment cultures 12 and 24 h post-treatment (Table 3). Additionally, *ODC* expression was decreased (*p* = 0.04) 1 h post-treatment in the fused cultures treated with Spe when compared to the control cultures, but *ODC* expression 12 and 24 h post-treatment was not different (*p* > 0.10) from the fused control cultures (Table 3). The treatment of fused cells with TBA, E2, TBA + E2, Met, Put, or Spd had no effect (*p* > 0.10) on *ODC* abundance compared to the control cultures at 1, 12, or 24 h post-treatment (Table 3). Analysis of *AMD1* expression showed that the treatment of fused cells with TBA tended (*p* = 0.09) to increase *AMD1* expression 12 h post-treatment when compared to the control cultures; however, no differences (*p* > 0.10) between the treatment and control cultures were observed 1 or 24 h post-treatment (Table 3). The reatment of fused cultures with Put resulted in increased (*p* = 0.002) *AMD1* expression 12 h after treatment compared to the control cultures (Table 3). No differences (*p* > 0.10) in *AMD1* expression between the fused control cultures and treatment cultures were observed 1 or 24 h post-treatment with Put (Table 3). The treatment of fused cells with E2, TBA + E2, Met, Orn, Spd, or Spe had no effect (*p* > 0.10) on *AMD1* abundance compared to the control cultures at 1, 12, or 24 h post-treatment (Table 3).

### 3.4. Effects of Treatments on the Relative mRNA Abundance of Genes Involved in Skeletal Muscle Growth

The treatment of proliferating C2C12 and Sol8 cells with Spd resulted in an increased (*p* = 0.03) relative mRNA abundance of *Pax7* 12 h post-treatment compared to the control cultures; however, no differences (*p* > 0.10) in *Pax7* abundance were observed 0.5 or 24 h post-treatment when compared to the control cultures (Table 4). Additionally, the relative mRNA abundance of *Pax7* was increased (*p* = 0.001) 24 h post-treatment when the proliferating cells were treated with Put, relative to the control cultures, but no differences (*p* > 0.10) in *Pax7* abundance in the proliferating cultures 0.5 or 12 h post-treatment were observed compared to the control cultures (Table 4). No differences (*p* > 0.10) in the relative mRNA abundance of *Pax7* were observed at 0.5, 12, or 24 h post-treatment when the proliferating cells were treated with TBA, Met, Orn, or Spe when compared to the control cultures (Table 4). Analysis of the relative mRNA abundance of *Spry1* showed that the proliferating cells treated with Put tended (*p* = 0.09) to have an increased relative mRNA abundance of *Spry1* 12 h post-treatment relative to the control cultures (Table 4). However, the treatment with Put in the proliferating cells had no effect (*p* > 0.10) on *Spry1* expression 0.5 or 24 h post-treatment when compared to the control cultures (Table 4). The relative abundance of *Spry1* in the proliferating cultures was also increased (*p* = 0.03) 24 h after the treatment with Orn when compared to the proliferating control cultures, but no differences (*p* > 0.10) in *Spry1* expression in the proliferating cells were observed 0.5 or 12 h post-treatment relative to the control cultures (Table 4). The relative mRNA abundance of *Spry1* at 0.5, 12, and 24 h post-treatment was not different (*p* > 0.10) in the proliferating cultures treated with TBA, Met, Spd, or Spe when compared to the proliferating control cultures (Table 4). The treatment of proliferating cells with Met resulted in a decreased (*p* = 0.03) mRNA abundance of *MapK* 0.5 h after treatment and an increased (*p* = 0.02) abundance 24 h after treatment when compared to the control cultures; however, no differences (*p* > 0.10) were observed 12 h post-treatment (Table 4). The relative mRNA abundance of *MapK* in the proliferating cultures was increased (*p* = 0.02) 24 h after treatment with Put when compared to the control cultures (Table 4), but no differences (*p* > 0.10) were observed 0.5 or 12 h post-treatment (Table 4). No differences (*p* > 0.10) in the relative mRNA abundance of *MapK* at 0.5, 12, or 24 h post-treatment were observed when the proliferating cells were treated with TBA, Orn, Spd, or Spe (Table 4).

The expression of *mTOR* tended (*p* = 0.08) to be decreased 1 h after treatment in the fused cultures treated with E2 compared to the control cultures; however, no differences (*p* > 0.10) were observed at 12 or 24 h post-treatment (Table 4). The treatment of the fused cultures with Orn decreased (*p* = 0.008) *mTOR* abundance 1 h after treatment compared to the control cultures (Table 4). No differences (*p* > 0.10) in *mTOR* abundance were observed between the control cultures and cultures treated with Orn 12 or 24 h post-treatment (Table 4). The abundance of *mTOR* in the fused cultures was increased (*p* = 0.007) 12 h post-treatment in the Put-treated cultures relative to the control cultures; however, no differences (*p* > 0.10) were observed 1 or 24 h after treatment (Table 4). The treatment of the fused cultures with Spd resulted in increased (*p* = 0.004) *mTOR* expression 12 h post-treatment compared to the control cultures, but no differences (*p* > 0.10) were observed at 1 or 24 h post-treatment (Table 4). The relative mRNA abundance of *mTOR* tended (*p* = 0.07) to be decreased 12 h after treatment in the fused cultures treated with Spe compared to the control cultures (Table 4). However, the treatment of cultures with Spe had no effect (*p* > 0.10) on *mTOR* mRNA abundance 1 or 24 h post-treatment (Table 4). No differences (*p* > 0.10) in *mTOR* abundance between the control cultures and cultures treated with TBA, TBA + E2, or Met were observed 1, 12, or 24 h post-treatment (Table 4).

Analysis of *eIF-2Bε* abundance showed that the treatment of cultures with Put increased (*p* = 0.01) *eIF-2Bε* abundance 12 h post-treatment relative to the control cultures; however, no differences (*p* > 0.10) were observed 1 or 24 h post-treatment (Table 4). Additionally, the treatment of cultures with Spe tended (*p* = 0.07) to increase *eIF-2Bε* abundance 12 h post-treatment but had no effect (*p* > 0.10) on *eIF-2Bε* abundance 1 or 24 h post-treatment (Table 4). No differences (*p* > 0.10) in *eIF-2Bε* expression were observed 1, 12, or 24 h after the treatment with TBA, E2, TBA + E2, Met, Orn, or Spd (Table 4).

## 4. Discussion

Androgens are potent stimulators of skeletal muscle growth and are known to enhance cell proliferation [19,21], differentiation [19], and protein synthesis [18,23]. Androgens have become hormones of interest in both humans and livestock species due to their known positive impacts on skeletal muscle growth. In humans, testosterone has been utilized as a treatment for conditions resulting in muscle atrophy, such as sarcopenia [12,13]. Trenbolone acetate is an androgenic compound that has 3–5 times the androgenic activity and 5–8 times the anabolic activity of testosterone [45,46] and is widely used in anabolic implants for cattle to improve growth and efficiency [14]. However, the exact mechanisms through which androgens improve skeletal muscle growth is not fully understood [45,47]. Additionally, over 50% of the consumer population is concerned with exogenous hormones being provided to beef cattle [48]. Previous research in mice [24], chickens [25], and rats [26,27,28] has shown that androgens interact with the polyamine biosynthetic pathway. An improved understanding of the interaction between androgens, the polyamine biosynthetic pathway, and growth, is necessary for the development of alternative ways to increase skeletal muscle growth. In the present study, the relationship between anabolic hormones, polyamines and polyamine precursors, and the mRNA abundance of genes involved in the polyamine biosynthesis pathway and skeletal muscle growth were examined in proliferating and fused C2C12 and Sol8 murine myoblast cultures.

To the best of the authors’ knowledge, no other studies have examined the differences in proliferation, protein synthesis, or the mRNA abundance of the genes measured between C2C12 and Sol8 murine myoblast cells. Although both the C2C12 and Sol8 cell lines are murine myoblasts, these cell lines grow at different rates because they are obtained from different types of skeletal muscle tissue and from animals at different stages of growth. The C2C12 cells are immortalized, non-cancerous murine myoblast cells obtained from muscle sarcomas of adult mice and Sol8 cells are myoblast cells obtained from the skeletal muscle of 4-week-old mice. As such, both cell lines were used in the present study to determine if treatment with anabolic hormones, polyamines, or polyamine precursors affects proliferation, protein synthesis, and gene expression of different types of myoblasts in a similar fashion. The present study demonstrates that the C2C12 and Sol8 cells respond similarly when treated with anabolic hormones, polyamine precursors, or polyamines. Utilization of both cell types in future studies may help to improve the power of cell culture experiments by examining two different clonal lines.

Increased proliferation rates were observed in the murine myoblasts treated with TBA, polyamine precursors, or polyamines compared to the control cultures. Polyamines are naturally occurring amino acid derivatives that are important modulators of growth, cell proliferation, and cell differentiation [24,32,33,34,35,36]. Putrescine, Spd, and Spe are produced from Met, Orn, and Arg through the polyamine biosynthetic pathway [24,32,33]. Androgenic compounds, such as TBA, are thought to regulate polyamine biosynthesis through increasing the expression of *ODC* and *AMD1* [39,40]. The positive effect of TBA on the proliferation rate of bovine satellite cells in culture has been well-established [21,22,42]. However, past work in C2C12 cells contrasts with the results observed in the present study and demonstrates that treatment with testosterone or dihydrotestosterone has no effect on proliferation rates [20,48], likely because C2C12 cells only express 0.1% of the androgen receptor mRNA of that found in the skeletal muscle of adult mice [48]. As such, the increased proliferation rates observed in C2C12 cells treated with TBA in the present study were likely due to TBA having increased androgenic and anabolic activity relative to testosterone [49]. Additionally, the effects of polyamines on proliferation rates of C2C12 cells from previous studies are inconsistent. Contrary to the observed results, a recent study found that the treatment of C2C12 cells with Put had no effect on proliferation rates 24, 48, 72, or 96 h post-treatment, but increased differentiation [50], while another study observed similar results to the present study and found that the treatment of C2C12 cells with concentrations of Spe that were less than those used in the present study (200, 600, or 2000 nM) increased proliferation rates 48 h post-treatment [51], indicating that Spe has a potent effect on proliferation. Additionally, the polyamine depletion of mouse fibroblasts cells results in an arrest of cell proliferation [52], demonstrating the importance of polyamines for proliferation. This study supports previous studies and suggests that TBA, polyamine precursors, and polyamines improve skeletal muscle growth by stimulating the proliferation of muscle precursor cells.

Growth of skeletal muscle occurs when protein synthesis rates exceed protein degradation rates [8]. In the present study, the treatment of C2C12 and Sol8 cells with TBA + E2 increased protein synthesis; however, the treatments with TBA, E2, Met, Orn, Put, Spd, or Spe had no effect on protein synthesis rates. In contrast to the results of the present study, previous research has shown that the treatment of C2C12 cells with 10 nM TBA results in increased protein synthesis rates [53]. Similar to the present study, the treatment of C2C12 cells with testosterone, dihydrotestosterone, or estradiol did not affect protein synthesis rates [20]. Androgen treatment alone likely does not affect the protein synthesis rates of C2C12 cells because they express androgen receptor mRNA at only 0.1% of the level found in muscle from adult mice [48]. However, previous work suggests that C2C12 cells express the estrogen receptor at levels similar to that of the uterus and ovary [54]. The positive effects of anabolic hormones on protein synthesis rates in culture has been well-characterized in bovine satellite cells and demonstrates that treatment with 10 nM TBA or 10 nM E2, the same concentrations used in the present study, results in increased protein synthesis rates [23,55], which was contrary to the results observed in the present study. To the best of the authors’ knowledge, the present study is the first to examine the effects of polyamines and their precursors on the protein synthesis rates of C2C12 and Sol8 murine myoblasts. Although the treatment of C2C12 and Sol8 myoblasts with polyamines and polyamine precursors did not result in increased protein synthesis rates in the present study, others have shown that depletion of polyamines with L-α-Difluoromethylornithine, an irreversible suicide inhibitor of *ODC* [56], results in decreased protein synthesis in NIH3T3 mouse fibroblasts through inhibition of translation initiation [52], further highlighting the importance of polyamines in protein synthesis. The results of the present study demonstrate that anabolic hormones increase protein synthesis rates in both bovine satellite cells and murine myoblasts. Further, polyamines and their precursors do not alter protein synthesis in C2C12 and Sol8 cells, but polyamines play a role in protein synthesis through the regulation of translation initiation and elongation, as demonstrated by others [52].

ODC is an enzyme involved in polyamine biosynthesis [39,40] through the production of Put from Orn [24]. Androgens are thought to directly modulate the polyamine biosynthesis pathway through the upregulation of *ODC* [39]. The present study analyzed the effects of anabolic hormones, polyamines, and polyamine precursors on the mRNA abundance of *ODC* and found that the treatment of proliferating and fused cultures with anabolic hormones had no effect on the mRNA abundance of *ODC*. The *ODC* gene promotor contains an androgen response element [57], and when the androgen receptor is knocked out in mice, the *ODC* mRNA abundance is decreased [58]. Contrary to the present study, previous research in mouse skeletal muscle tissue shows that treatment with a selective androgen receptor modulator, a therapeutic compound that has anabolic effects similar to that of anabolic steroids without having the androgenic characteristics, results in an increased mRNA expression of *ODC* 14 d after treatment [51], which provides further evidence that an interaction between androgens and the polyamine biosynthetic pathway exists through *ODC*. However, the abundance of *ODC* in the proliferating cultures was unaffected by the treatment with polyamine precursors and was increased when cells were treated with Spe or Spd 0.5 and 12 h after treatment. In fused cells, the treatment of cultures with polyamine precursors showed that *ODC* expression tended to be decreased 1 h after treatment with Orn compared to the control cultures, however, treatment with Met did not affect *ODC* abundance in fused cells. Polyamine concentration is known to be tightly regulated within the cell and *ODC* activity is subject to negative feedback regulation by increased polyamine concentration [59]. These data suggest that the treatment of murine myoblasts with Spd and Spe at the concentrations used in the present study increases *ODC* expression, which may play a role in the increased proliferation rates observed in cells that were treated with polyamines, but that treatment with TBA or polyamine precursors does not impact the mRNA abundance of *ODC* in proliferating cells at the time points measured. Furthermore, the treatment of fused murine myoblasts with anabolic hormones does not impact *ODC* mRNA abundance; however, treatment with polyamines and their precursors may have an inhibitory effect on *ODC*, as treatment with these molecules has the potential to in decrease *ODC* expression.

Another important enzyme involved in the polyamine biosynthetic pathway is *AMD1* [39,40], which is involved in the production of decarboxylated S adenosylmethionine from adenosylmethionine [24]. Decarboxylated S adenosylmethionine can then stimulate the production of Spd and Spe through spermidine synthase and spermine synthase, respectively [32]. In the present study, the expression of *AMD1* in proliferating cells was unaffected by any of the treatments given. However, the treatment of fused cultures with TBA tended to increase *AMD1* expression 12 h post-treatment and the treatment of fused cultures with Put increased *AMD1* expression 12 h after treatment. Previous studies have found that *AMD1* is likely a direct target of the androgen receptor [57] and when the androgen receptor is knocked out in mice, *AMD1* expression is decreased [58]. To the best of the authors’ knowledge, no other published studies have reported the effects of polyamines and their precursors on *AMD1* expression in proliferating and fused murine myoblast cells. Overall, the results of the present study indicate that treatments of murine myoblasts with TBA, polyamines, or polyamine precursors do not affect *AMD1* expression in proliferating cells at the time points measured, but the treatment of fused murine myoblasts with TBA and Put has the potential to increase *AMD1* expression 12 h after treatment.

Quiescent muscle satellite cells express *Pax7* [3] and, once activated and committed to the myogenic lineage, *Pax7* decreases. Past work has shown that C2C12 cells still express *Pax7*, but at lower abundance than satellite cells [60]. In the present study, the treatment of C2C12 and Sol8 cells with Spd or Put resulted in an increased relative abundance of *Pax7* 12 and 24 h post-treatment. Past work has demonstrated that the treatment of C2C12 cells with the androgenic compound dihydrotestosterone results in increased proliferation and differentiation, as well as increased *Pax7* protein expression in differentiating cells 2 and 4 d after treatment and decreased *Pax7* expression 6 and 8 d after treatment [19], indicating that, as C2C12 cells differentiate, *Pax7* expression decreases. In contrast to the results of the present study, a previous study found that *Pax7* expression was not affected when proliferating bovine satellite cells were treated with polyamines, but observed increased *Pax7* expression when treated with Met [22]. Perhaps, increased Put concentrations are contributing to a negative feedback mechanism of *Pax7* transcription. Furthermore, the increased *Pax7* abundance observed in myoblast cells treated with Spd and Put in the present study demonstrates that more myoblasts may be present at 12 and 24 h post-treatment or that these myoblasts may be proliferating to replenish the satellite cell pool. However, more research needs to be completed to further determine how the increased *Pax7* abundance in proliferating myoblasts impacts myoblast growth.

Sprouty1 is necessary for the renewal of the quiescent satellite cell population and is a marker of decreased satellite cell proliferation [3,4]. To further examine the effects of TBA, polyamines, and polyamine precursors on genes related to cell growth, the expression of *Spry1* was also analyzed. The treatment of cells with Put tended to increase *Spry1* expression 12 h after treatment. Additionally, the expression of *Spry1* was increased when cells were treated with Orn 24 h after treatment. Recent work completed in bovine satellite cells observed that *Spry1* expression was increased 12 h post-treatment with TBA compared to control cultures [22], suggesting that the treatment of bovine satellite cells with TBA results in a decreased proliferation of satellite cells and the return of satellite cells to the quiescent state. The results observed in the present study combined with the results from past work suggest that TBA stimulates the return of activated satellite cells to quiescence 12 h after treatment, but likely results in satellite cell activation and progression down the myogenic lineage prior to 12 h post-treatment. Overall, these results, which align with the findings from analysis of *Pax7* expression, suggest that adequate concentrations of polyamines and their precursors in the cell may promote increased *Spry1* expression to, perhaps, prevent myoblasts from differentiating and promote proliferation instead.

Myoblasts are capable of proliferating, as marked by increased expression of *MapK* [7] and can eventually fuse into existing muscle fibers to increase muscle fiber hypertrophy and, ultimately, promote skeletal muscle growth [5,6]. In the present study, the analysis of *MapK* expression showed that the treatment of cells with Met decreased *MapK* expression 0.5 h after treatment and increased the expression of *MapK* 24 h after treatment when compared to the control cultures. The expression of *MapK* was increased 24 h after treatment with Put and was not affected by the treatments with TBA, Orn, Spd, or Spe. These results indicate that an increase in *MapK* abundance plays a key role in the increased proliferation rates of cells treated with Met and Put. However, the response of cells to these molecules is delayed until 24 h after treatment. The delay of *MapK* upregulation is likely contributing to the increased proliferation rates observed in the cultures treated with Met or Put. Of note, the primary regulation of MapK activity is through phosphorylation and dephosphorylation of the protein [61]. In contrast to the present study, previous research has shown that treatment of C2C12 cells with 10 nM TBA promoted the MapK pathway through increased extracellular signal-regulated kinase 1/2 (*ERK1/2*) phosphorylation [54]. Furthermore, treatment of L6 rat myoblast cells with testosterone increases proliferation through the MapK pathway by increasing *ERK* activity 5 to 20 min post-treatment, but *ERK* activity has been shown to decline 20 min post-treatment [62]. Together, results from previous studies suggest that one of the mechanisms through which androgens increase cell proliferation is through non-genomic mechanisms involving the MAPK/ERK pathway. The increased proliferation rates observed in cells treated with Orn, Spd, and Spe must occur through mechanisms other than the upregulation of *MapK*, or different time points need to be investigated to determine the time frame at which this occurs.

mTOR is a serine/threonine kinase that modulates protein synthesis through the activation of S6 kinase and inhibition of 4E-binding protein 1 [9,63]. The present study examined the effects of anabolic hormones, polyamine precursors, and polyamines on the abundance of *mTOR*. The present study found that the abundance of *mTOR* tended to be decreased 1 h after treatment with E2; however, no differences were observed in the cultures treated with TBA or TBA + E2. In contrast to the results of the present study, previous studies have observed increased mammalian target of rapamycin complex 1 (*mTORC1*) activity and *mTOR* phosphorylation after C2C12 cells were treated with testosterone or TBA, respectively [64]. The analysis of polyamine precursors and their effects on *mTOR* expression demonstrated that treatment with Orn decreases *mTOR* expression 1 h after treatment, but that *mTOR* expression is unaffected by treatment with Met, which contrasted previous work that observed increased *mTORC1* activity in murine embryonic fibroblasts and human embryonic kidney 293A cells 15 min after treatment with Met [46]. The treatment of cultures with polyamines resulted in an increased *mTOR* expression 12 h after treatment with Put and Spd and a tendency for *mTOR* abundance to be increased 12 h after treatment with Spe. In agreement with the results observed in the present study, previous studies have shown that Spd and Spe stimulate *mTORC1* activity in rat intestinal epithelial cells [65]. Overall, these results indicate that treatment of murine myoblasts with E2 or Orn may decrease *mTOR* expression, while treatment with polyamines increases *mTOR* abundance. As such, further research is needed to determine the exact effects of alterations in *mTOR* expression from treatment with these molecules.

*eIF-2Bε* is another gene that is important in protein synthesis [10,66]. In an anabolic state (i.e., during protein synthesis), expression of *eIF-2Bε* is increased in rat fibroblast cells and rat skeletal muscle [67,68,69,70]. *eIF-2Bε* initiates translation through the protein kinase B (AKT/PKB) pathway and is independent of *mTOR* [10]. In short, AKT/PKB phosphorylates glycogen synthase kinase-3 (GSK-3), inhibiting GSK-3 from phosphorylating (inhibiting) *eIF-2Bε* [10,71]. In the present study, the treatment with anabolic hormones or polyamine precursors had no effect on *eIF-2Bε* expression. However, the treatment with Put increased the expression of *eIF-2Bε* and the treatment with Spe tended to increase *eIF-2Bε* abundance 12 h after treatment. Although the treatment of cultures with Met in the present study did not result in increased *eIF-2Bε* expression, eukaryotic translation initiation factor 2B is known to play a role in mRNA translation and, in the presence of amino acids, has been shown to promote skeletal muscle hypertrophy in human embryonic kidney cells through repression of *eIF-2Bε* phosphorylation [10,72]. Together, these data suggest that polyamines may interact with the AKT/PKB pathway to increase *eIF-2Bε* abundance. However, it is important to note that eIF-2Bε is also largely regulated at the translational and post-translational levels [73]. As such, further research that examines how the treatments used in the present study affect eIF-2Bε protein expression and/or post-translational modifications is warranted.

## 5. Conclusions

Overall, the present study found that the treatment of proliferating and fused murine myoblast cultures with anabolic hormones increases proliferation and protein synthesis rates and treatment with polyamine precursors and polyamines increase proliferation rates, while having no effect on protein synthesis rates. Analysis of mRNA abundance showed that the treatment of proliferating murine myoblasts with polyamines increases mRNA expression of genes involved in polyamine biosynthesis and growth and polyamines and their precursors may function as signaling molecules to prevent the differentiation of myoblasts, alternatively promoting proliferation. Additionally, the treatment of proliferating cultures with polyamines increased *ODC* abundance, while *AMD1* abundance was unaffected by treatment. Analysis of the genes involved in skeletal muscle growth demonstrated that the treatment of proliferating cultures with polyamines resulted in an increased abundance of *Pax7* and *MapK* and tended to increase *Spry1* expression and the treatment of proliferating cultures with polyamine precursors resulted in an increased expression of *Spry1* and both increased and decreased *MapK* expression depending on the time point. The treatment with TBA had no effect on the mRNA abundance of genes involved in polyamine biosynthesis or skeletal muscle growth in proliferating cultures. Additionally, analysis of the mRNA abundance of genes involved in polyamine biosynthesis in fused cultures showed that treatment of cultures with anabolic hormones decreases *ODC* abundance and treatment with polyamines increases *AMD1* abundance. Assessment of the genes involved in protein synthesis showed that *mTOR* abundance was decreased after the treatment with anabolic hormones and polyamine precursors and increased after the treatment with polyamines, while *eIF-2Bε* expression was increased after the treatment with polyamines and unaffected by the treatment with polyamine precursors and anabolic hormones. Ultimately, additional research is needed to determine the effects of anabolic hormones, polyamine precursors, and polyamines on a wider array of mRNA targets and at more time points. An improved understanding of androgens and their effect on the polyamine biosynthetic pathway and growth is essential for the development of natural alternatives to improve and/or enhance skeletal muscle growth.

## Figures and Tables

**Table 1:** Primer and Probe Sequences used in real-time qPCR for analysis of mRNA expression in proliferating and fused murine myoblast cells.

Messenger RNA	GBA Number	Primer and Probe Sequences (5′-3′)
Ribosomal 18S (*18S*)	AF243428	FP: CCACGCGAGATTGAGCAAT RP: GCAGCCCCGGACATCTAA TP: ACAGGTCTGTGATGCC
Ornithine decarboxylase (*ODC*)	NM_013614.2	FP: CCTGAGCGGATGAGCATTATAG RP: CGACAGACAGCTTTGGAATCA TP: AGGTTGGTTCTACGGATTGCCACT
S-adenosylmethionine decarboxylase (*AMD1*)	NM_009665.5	FP: CTACTTGTCCTACCGTCAGCTG RP: CAGAATATTGCGCCGTTCCATC TP: CAGGTTACTCAGCCAGATAGTGAA
Paired box transcription factor 7 (*Pax7*)	XM_616352.4	FP: TTGTACCCCGCCCTCTCTTA RP: GGCTCAGCAATCCGTTTCC TP: AGCTGGGTCTTTTG
Sprouty 1 (*Spry1*)	NM_001099366.1	FP: TCACAGGAAGACAGCAAAGA RP: GCAAACAGGAAGACACGAC TP: TGCTTCTTAGAAGCTGGAGAGCA
Mitogen-activated protein kinase (*MapK*)	NM_001038663.1	FP: CCACCCATACCTGGAGCAGTA RP: CAAACTTGAATGGCGCTTCA TP: CCCAAGTGATGAGCCCA
Eukaryotic translation initiation factor 2B subunit epsilon (*eIF-2Bε*)	NM_172265.2	FP: CAAAGAGACACAACTGACGAAGG RP: GTTACGAGGACAGCCAATGAGA TP: CTGAGAGAGGCAGAAGAAGAGTC
Mammalian target of rapamycin (*mTOR*)	NM_020009.2	FP: CATCCCTCTGTCCACCAACTC RP: TGCTCAAACACCTCTACCTTCT TP: CGGGACTACAGAGAGAAGAAGAAG

GBA, gene bank accession; FP, forward primer; RP, reverse primer; TP, TaqMan probe.

**Table 2:** Relative mRNA abundance of *ODC*, *AMD1*, *Pax7*, *Spry1*, *MapK*, *mTOR*, and *eIF-2Bε* in proliferating and fused C2C12 and Sol8 cells at 0.5 and 1, 12 and 24 h after treatment.

**Proliferating cultures ^a^**				
Time (h)	C2C12	Sol8	SEM	*p*-value
*ODC*				
0.5	1.23	0.96	0.19	*p* = 0.32
12	0.97	1.13	0.23	*p* = 0.63
24	1.04	0.86	0.37	*p* = 0.56
*AMD1*				
0.5	1.38	1.54	0.43	*p* = 0.79
12	1.03	1.02	0.18	*p* = 0.95
24	1.22	1.01	0.27	*p* = 0.57
*Pax7*				
0.5	1.10	0.84	0.30	*p* = 0.52
12	1.08	0.74	0.15	*p* = 0.10
24	1.37	0.88	0.25	*p* = 0.14
*Spry1*				
0.5	1.13	1.10	0.24	*p* = 0.91
12	0.99	0.92	0.21	*p* = 0.89
24	1.43	0.85	0.30	*p* = 0.14
*MapK*				
0.5	0.96	0.78	0.09	*p* = 0.17
12	0.96	0.92	0.21	*p* = 0.89
24	1.43	0.85	0.30	*p* = 0.14
**Fused cultures ^b^**				
Time (h)	C2C12	Sol8	SEM	*p*-value
*ODC*				
1	0.87	0.81	0.14	*p* = 0.77
12	1.13	1.3	0.15	*p* = 0.42
24	0.87	1.31	0.25	*p* = 0.22
*AMD1*				
1	1.10	0.65	0.17	*p* = 0.06
12	1.50	1.59	0.47	*p* = 0.89
24	0.85	0.96	0.16	*p* = 0.61
*mTOR*				
1	0.87	0.68	0.11	*p* = 0.22
12	1.30	1.41	0.22	*p* = 0.70
24	0.88	1.38	0.21	*p* = 0.10
*eIF-2Bε*				
1	0.95	0.91	0.19	*p* = 0.88
12	1.05	1.28	0.13	*p* = 0.20
24	0.90	1.32	0.21	*p* = 0.17

^a^ Proliferating cultures: Relative mRNA abundance of ornithine decarboxylase (*ODC*), s-adenosylmethionine decarboxylase (*AMD1*), paired box transcription factor 7 (*Pax7*), Sprouty1 (*Spry1*), and mitogen-activated protein kinase (*MapK*) in proliferating C2C12 (n = 3) and Sol8 (n = 3) murine myoblast cells, 0.5 h, 12 h, and 24 h after treatment as described in the Materials and Methods section. This table demonstrates differences in mRNA abundance between the two cell types, regardless of treatment. ^b^ Fused cultures: Relative mRNA abundance of *ODC*, *AMD1*, mammalian target of rapamycin (*mTOR*), and eukaryotic translation initiation factor 2B subunit epsilon (*eIF-2Bε*) in fused C2C12 (n = 5) and Sol8 (n = 5) murine myoblast cells, 1 h, 12 h, and 24 h after treatment as described in the Materials and Methods section. This table demonstrates differences in mRNA abundance between the two cell types, regardless of treatment.

**Table 3:** Effects of treatments on the relative mRNA abundance of genes involved in polyamine biosynthesis in proliferating and fused myoblast cells ^a^.

**Proliferating Cultures ^a^**										
	Treatment									
Time (h)	Con	TBA		Met	Orn		Put	Spd	Spe	SEM
*ODC*										
0.5	0.96	0.79		0.77	1.13		0.83	0.74	1.96 *	0.54
12	0.96	0.74		0.90	0.72		1.06	2.09 *	1.42	0.50
24	0.95	0.96		1.07	1.06		0.76	0.35	1.34	0.80
*AMD1*										
0.5	1.10	1.24		1.89	1.50		1.82	1.12	1.85	0.62
12	1.00	1.51		1.15	1.20		1.19	0.76	0.61	0.27
24	0.97	1.13		1.42	1.15		1.66	1.14	0.96	0.44
**Fused cultures ^b^**										
	Treatment									
Time (h)	Con	TBA	E2	TBA + E2	Met	Orn	Put	Spd	Spe	SEM
*ODC*										
1	1.00	0.75	0.98	0.98	0.75	**0.71 ^†^**	0.82	0.68	0.83	0.14
12	1.00	1.07	1.29	1.11	1.21	1.19	1.38	1.40	1.27	0.20
24	1.00	1.20	0.83	1.30	1.00	1.45	1.08	1.10	0.79	0.32
*AMD1*										
1	1.00	0.93	0.78	0.83	0.90	0.78	0.92	0.84	0.83	0.25
12	1.00	1.71 ^†^	1.65	1.46	1.44	1.64	2.31 *	1.71	1.14	0.45
24	1.00	1.05	0.84	1.05	0.96	0.88	1.15	0.83	0.42 *	0.17

^a^ Proliferating cultures: Relative mRNA abundance of ornithine decarboxylase (*ODC*) and S-adenosylmethionine decarboxylase (*AMD1*) in proliferating C2C12 and Sol8 murine myoblast cells 0.5 h, 12 h, and 24 h after treatment with 1% fetal bovine serum (FBS, control, Con), 10 nM trenbolone acetate (TBA), 10 mM methionine (Met), 8 mM ornithine (Orn), 3 mM putrescine (Put), 1.5 mM spermidine (Spd), or 0.5 mM spermine (Spe). ^b^ Fused cultures: Relative mRNA abundance of *ODC* and *AMD1* in fused C2C12 and Sol8 murine myoblast cells 1 h, 12 h, and 24 h after treatment with serum-free media (SFM, control, Con) as a control. The other treatments were similar to those described in the proliferation assay with the exception of the addition of 10 nM TBA + 10 nM E2 (TBA + E2). Values with a star (*) indicate a difference (*p* ≤ 0.05) whereas a cross (†) indicates a tendency (0.05 < *p* ≤ 0.10) for difference in relative mRNA abundance compared to control cultures within a time point.

**Table 4:** Effects of treatments on the relative mRNA abundance of genes involved in growth in proliferating and fused myoblast cells ^a^.

**Proliferating cultures ^a^**										
	Treatment									
Time (h)	Con	TBA		Met		Orn	Put	Spd	Spe	SEM
*Pax7*										
0.5	0.91	1.02		1.64		0.57	0.58	1.02	1.17	0.66
12	0.97	0.83		1.09		0.98	0.56	1.68 *	0.67	0.29
24	0.96	0.96		1.59		0.98	2.94 *	0.71	1.27	0.46
*Spry1*										
0.5	1.00	1.22		0.78		1.00	1.05	1.74	1.26	0.83
12	0.98	1.74		1.01		1.34	1.81 *	0.42	0.70	0.44
24	1.00	1.55		1.27		2.08 *	1.00	0.71	0.63	0.69
*MapK*										
0.5	1.00	0.84		0.20 *		0.75	0.66	0.96	1.03	0.53
12	1.00	0.70		1.32		0.86	1.08	0.62	0.76	0.33
24	0.93	1.08		1.96 *		0.53	2.09 *	0.71	1.49	0.47
**Fused cultures ^b^**										
	Treatment									
Time (h)	Con	TBA	E2	TBA + E2	Met	Orn	Put	Spd	Spe	SEM
*mTOR*										
1	1.00	0.84	0.71 ^†^	0.71	0.83	0.56 *	0.78	0.83	0.69 ^†^	0.15
12	1.00	1.20	1.20	1.45	1.08	1.10	1.87 *	1.97 *	1.31	0.27
24	1.00	0.33	1.04	1.45	1.30	1.34	0.79	1.20	0.96	0.33
*eIF-2Bε*										
1	1.00	0.97	0.86	1.02	0.90	0.90	0.91	0.95	0.91	0.18
12	1.00	0.98	1.04	1.21	1.07	1.13	1.44 *	1.28	1.31 ^†^	0.18
24	1.00	1.29	0.99	1.14	1.35	1.01	1.29	0.97	0.92	0.26

^a^ Proliferating cultures: Relative mRNA abundance of paired box transcription factor 7 (*Pax7*), Sprouty1 (*Spry1*), and mitogen-activated protein kinase (*MapK*) in proliferating C2C12 and Sol8 murine myoblast cells 0.5 h, 12 h, and 24 h after treatment with 1% fetal bovine serum (FBS, control, Con), 10 nM trenbolone acetate (TBA), 10 mM methionine (Met), 8 mM ornithine (Orn), 3 mM putrescine (Put), 1.5 mM spermidine (Spd), or 0.5 mM spermine (Spe). ^b^ Fused cultures: Relative mRNA abundance of mammalian target of rapamycin (*mTOR*) and eukaryotic translation initiation factor 2B subunit epsilon (*eIF-2Bε*) in fused C2C12 and Sol8 murine myoblast cells 1 h, 12 h, and 24 h after treatment with serum-free media (SFM, control, Con) as a control. The other treatments were similar to those described in the proliferation assay with the exception of the addition of 10 nM TBA + 10 nM E2 (TBA + E2). Values with a star (*) indicate a difference (*p* ≤ 0.05) whereas a cross (†) indicates a tendency (0.05 < *p* ≤ 0.10) for difference in relative mRNA abundance compared to control cultures within a time point.

## Data Availability

The data presented in this study are available on fair request from the respective author.
